# Effects of Fermentation Broth from the Biocontrol Fungus *Diaporthe novem* on *Colletotrichum jiangxiense*, the Causal Agent of Rhododendron Brown Spot, and Transcriptomic Analysis of the Pathogen

**DOI:** 10.3390/microorganisms14071530

**Published:** 2026-07-13

**Authors:** Mengyao Wang, Yajiao Sun, Huali Li, Jian Liu, Shuwen Liu, Ruiyan Pan, Yunqiang Ma, Junjia Lu

**Affiliations:** 1 College of Landscape Architecture and Horticulture Sciences, Southwest Forestry University Sciences, Kunming 650224, China; 17587020327@163.com (M.W.); 18087323192@126.com (Y.S.); 15912938064@163.com (H.L.); jian927520@163.com (J.L.); 15887642939@163.com (S.L.); pry20030403@163.com (R.P.); 2 Yunnan Key Laboratory of Forest Disaster Warning and Control, Southwest Forestry University, Kunming 650224, China; 3 Yunnan Key Laboratory of Landscape Plant Resource Cultivation and Application, Southwest Forestry University, Kunming 650224, China; mayunqiang@swfu.edu.cn

**Keywords:** rhododendron brown spot, biocontrol fungus, *Diaporthe novem*, *Colletotrichum jiangxiense*, antifungal mechanism, transcriptome analysis

## Abstract

The fungal pathogen *Colletotrichum jiangxiense* has a broad host range and high destructive potential. It is a major causal agent of brown spot disease in diverse plants. Antifungal mechanisms used by biocontrol fungi against plant pathogens include disruption of cellular structures and cell wall damage, which can lead to protoplast leakage and hyphal lysis. In this study, we investigated the antifungal mechanism of the endophytic fungus *Diaporthe novem* DJ13 against *C. jiangxiense*, the causal agent of rhododendron brown spot. DJ13 is an effective biocontrol strain previously isolated by our research group from healthy leaves of *Rhododendron pulchrum*. Physiological assays showed that treatment with DJ13 fermentation broth increased membrane permeability, elevated MDA content, reduced TCA cycle enzyme activities, and increased AKP activity. These findings suggest impaired membrane integrity, disrupted energy metabolism, and cell wall damage. Transcriptomic analysis of the treated pathogen identified 1680 significantly differentially expressed genes (DEGs), including 961 up-regulated and 719 down-regulated genes. Among these genes, ABC transporter genes were significantly up-regulated, whereas genes involved in membrane structure metabolism were significantly down-regulated. Chitinase genes were up-regulated, whereas α-glucanase genes were down-regulated. DASH family cryptochrome genes were significantly down-regulated, while genes related to reactive oxygen species (ROS) production, including xanthine dehydrogenase, were significantly up-regulated. In addition, FAD-dependent oxidoreductase genes were up-regulated, while respiratory-metabolism-related genes, including trimethyllysine dioxygenase, were down-regulated. Together, the physiological and transcriptomic data provide a correlative framework supporting the hypothesis that the antifungal mechanism of DJ13 fermentation broth may involve the coordinated action of four processes: cell membrane damage, cell wall disruption, oxidative stress, and inhibition of energy metabolism. These findings also identify candidate genes for future functional validation.

## 1. Introduction

*Rhododendron hybridum*, a woody ornamental plant in the family Ericaceae and genus Rhododendron, has high ornamental value and is often referred to in China as the “beauty among flowers” [1]. However, diseases associated with anthropogenic factors, insects, and microorganisms are highly prevalent in rhododendrons. These diseases can cause petal and leaf yellowing, wilting, reduced ornamental and medicinal value, and even plant death. Reported root diseases include stem rot, canker, root rot, and shoot dieback, whereas major leaf diseases include brown spot, gray mold, anthracnose, black spot, and leaf blight [2,3]. Among these diseases, brown spot is one of the most destructive during rhododendron cultivation and severely impairs plant growth and development. Tian et al. [4] investigated the occurrence of brown spot in the Kunming area of Yunnan Province and found that it poses a serious threat to *R. hybridum* growth. Under open-field cultivation, the disease usually appears between April and May and can persist until December. Under conditions of high temperature, high humidity, and abundant rainfall, older leaves are infected first and may serve as a major source of disease epidemics.

The genus *Colletotrichum* has a broad host range and wide geographic distribution and has been widely studied. For example, *C. fructicola* causes brown spot on cactus in Brazil [5], while *C. gloeosporioides* causes this disease on grapes in Sri Lanka [6] and bottle tree in Argentina [7]. Brown spot symptoms caused by *C. jiangxiense* are characterized by small brown lesions that gradually coalesce into contiguous scorched patches. This pattern of disease progression is similar to that caused by other *Colletotrichum* species. These findings support the host adaptability and symptom diversity of this genus and are consistent with the reported diversity of rhododendron leaf diseases [8,9].

Currently, rhododendron brown spot is controlled mainly through chemical management, supplemented by agricultural practices [10]. However, prolonged use of conventional chemical fungicides can promote pathogen resistance, cause environmental pollution, and disrupt ecological balance. These limitations underscore the need for effective alternative strategies. Biological control has several advantages, including high specificity, environmental safety, and a low risk of resistance development. Among biocontrol agents, plant endophytic fungi colonize plant tissues asymptomatically and can enhance host resistance to disease [11,12]. Previous studies have shown that endophytes can be more effective than chemical agents in controlling pathogen infection. For example, Mei et al. [13] reported that an endophytic bacterial inoculant controlled strawberry diseases during the fruiting period more effectively than chemical fungicides.

Endophytic fungi can suppress pathogen growth by secreting antimicrobial compounds and competing for ecological niches. Their antifungal effects are mediated mainly through disruption of cell membrane integrity and degradation of the cell wall. Consistently, Macías-Rubalcava et al. [14] and Luo et al. [15] demonstrated that crude extracts from endophytic fungi inhibited pathogenic fungi by compromising membrane integrity and interfering with normal metabolism. Chen [16] found that 2-nonanone, an active metabolite from strain BV6, caused hyphal collapse, growth retardation, increased reactive oxygen species (ROS) levels, and impaired membrane integrity in *Botrytis cinerea*. Peng et al. [17] reported that crude extracts of *Bacillus altitudinis* isolated from tea leaves increased cell membrane permeability, electrical conductivity, and soluble sugar and MDA contents in pathogenic fungal mycelia, while reducing soluble protein content.

Advances in mRNA enrichment and high-throughput sequencing have greatly improved the identification of functional genes in microorganisms. Transcriptomic studies of pathogenic fungi exposed to biocontrol agents have shown that these agents can induce extensive changes in pathogen gene expression. The resulting differentially expressed genes (DEGs) are involved in a wide range of biological processes, including cell wall biosynthesis, antioxidant defense, metabolism, and plant–pathogen interactions. Although pathogenic fungi frequently activate stress and defense responses, these responses are often insufficient to prevent cellular injury. Biocontrol fungi may therefore exert antifungal activity by reshaping pathogen transcriptional programs and disrupting essential cellular processes.

Several studies have examined the transcriptional responses of fungal pathogens to bacterial biocontrol agents. Zhang et al. [18] isolated *Bacillus velezensis* YB-185 from wheat rhizosphere soil and demonstrated its strong antagonistic activity against *Fusarium pseudograminearum*. Transcriptomic analysis of *F. pseudograminearum* co-cultured with YB-185 showed that the pathogen attempted to preserve cellular function by increasing cell wall and membrane synthesis, activating antioxidant and stress-response pathways, detoxifying antimicrobial compounds, and transporting damaged cellular components. Nevertheless, cell death and free radical accumulation continued to occur, indicating that these compensatory responses did not prevent cellular damage. Li et al. [19] analyzed the transcriptomic response of *Botrytis cinerea* to a crude extract of *Bacillus velezensis* HY19. The extract induced broad transcriptional changes, most of which involved down-regulated genes associated with biological processes, cellular components, and molecular functions in *B. cinerea*. Similarly, Xu et al. [20] isolated the endophytic bacterium *Bacillus velezensis* QSE-21 from tomato stems and showed that it significantly inhibited *B. cinerea*. Transcriptomic analysis further indicated that genes involved in metabolic processes were differentially expressed.

Among fungal biocontrol agents, Azeez et al. [21] co-cultured the endophytic fungus *Simplicillium lamellicola* with the pathogenic fungus *Thielaviopsis paradoxa* and showed that this mycoparasitic biocontrol agent induced complex defense responses in the pathogen. These responses included altered expression of genes related to cell-wall-degrading enzymes and oxidative stress. Pimentel et al. [22] employed dual RNA-seq to examine the interaction between *Trichoderma afroharzianum*, a species closely related to *T. harzianum*, and *Fusarium virguliforme*, the causal agent of soybean sudden death syndrome. Their results showed that the pathogen displayed marked transcriptomic plasticity in response to different Trichoderma isolates. Genes encoding CAZymes and CBM1 domain-containing proteins were up-regulated before physical contact, suggesting that volatile compounds may contribute to early recognition between the two fungi. Lysøe et al. [23] performed a three-party transcriptomic analysis involving *Clonostachys rosea*, the potato pathogen *Helminthosporium solani*, and the potato host. During interaction with the pathogen, genes associated with oxidative stress responses, proteases, and G protein signaling were significantly up-regulated in *C. rosea*, whereas pathogen gene expression was strongly suppressed. These findings suggest that *C. rosea* mediates biocontrol through several coordinated mechanisms, including mycoparasitism, activation of host defense responses, and nutrient competition. Feng et al. [24] co-cultured *Verticillium dahliae* Vd076 with the culture filtrate of the endophytic fungus CEF08111 and performed transcriptome sequencing. At 3 h post-inoculation, DEGs were mainly enriched in carbohydrate metabolism and phenylpropanoid biosynthesis pathways. Atanasova et al. [25] analyzed *Trichoderma harzianum* co-cultured with *Rhizoctonia solani* and found that Trichoderma promoted pathogen cell wall degradation by up-regulating chitinase and glucanase genes. Xu et al. [26] further showed that the fermentation filtrate of *Purpureocillium lilacinum* induced transcriptional reprogramming of genes related to energy metabolism and cell wall degradation in nematode egg-parasitic fungi. Together, these findings indicate that fungal biocontrol agents inhibit pathogen growth through multiple interacting mechanisms, including cell wall degradation, oxidative stress induction, and disruption of energy metabolism.

Rhododendron brown spot is a serious disease affecting the rhododendron industry, yet the biological control of this disease using endophytic fungi remains poorly studied. In the present study, *Colletotrichum jiangxiense*, the causal agent of brown spot on *Rhododendron hybridum*, and the endophytic fungal strain DJ13 (*Diaporthe novem*) were used as experimental materials. DJ13 was selected from eight candidate strains isolated from healthy tissues of *Rhododendron pulchrum*. The selection was based on a comprehensive evaluation of antifungal inhibition rate, growth-promoting capacity, growth-promoting effects in potted plants, and cell-wall-degrading enzyme activities. Among the eight candidates, DJ13 showed the strongest biocontrol activity against *C. jiangxiense*, with an inhibition rate of 66.23%. The growth-promoting potential of the candidate endophytic fungi was assessed by measuring phosphate solubilization, potassium solubilization, siderophore production, and indole-3-acetic acid (IAA) production. DJ13 showed a phosphate-solubilizing rate of 35.12%, a potassium-solubilizing capacity of 103.19 mg/L, a siderophore production value of 34.62% in the chrome azurol S assay, and an IAA production level of 10.04%. Rhododendron seedlings treated with DJ13 performed better than seedlings treated with the other strains across all measured growth indicators, including plant height, root length, stem diameter, leaf area, chlorophyll content, and biomass. DJ13 also showed the highest cell-wall-degrading enzyme activities among the candidate strains. Its cellulase, β-1,3-glucanase, chitinase, protease, and amylase activities reached 481.99 U/mL, 214.08 U/mL, 126.76 U/mL, 861.91 U/mL, and 481.99 U/mL, respectively, and were significantly higher than those of the other strains. To investigate the antifungal mechanism of DJ13, its fermentation broth was co-cultured with *C. jiangxiense*. The effects of the fermentation broth on cell membrane integrity and protein metabolism were then evaluated. Transcriptomic analysis of the treated pathogen was performed to identify molecular responses associated with DJ13-mediated antifungal activity. This study provides a microbial resource for the biological control of rhododendron brown spot and offers a theoretical basis for the development and application of endophytic fungal biocontrol agents.

## 2. Materials and Methods

### 2.1. Experimental Materials

#### 2.1.1. Experimental Strains

*Colletotrichum jiangxiense*, used as the pathogen in this study, was originally isolated from diseased leaves of *Rhododendron hybridum* exhibiting typical brown spot symptoms. The isolate was purified, and its pathogenicity was verified through inoculation assays on both detached leaves and intact plants, followed by morphological and molecular identification.

To obtain an effective biocontrol agent, eight endophytic fungal strains with significant inhibitory activity against *C. jiangxiense* were isolated from the stems and leaves of healthy *Rhododendron pulchrum* by our research group. These strains were subsequently subjected to comprehensive screening. Among the eight candidate strains, strain DJ13 showed the best overall performance and was identified as *Diaporthe novem*; it was therefore selected for all subsequent experiments. All strains are preserved in the laboratory of Southwest Forestry University.

#### 2.1.2. Experimental Media

Potato Dextrose Broth (PDB): 200 g potato, 20 g glucose, 1000 mL deionized water, pH 6.0–6.5.

### 2.2. Preparation of Fermentation Broth of Strain DJ13

Three 5-mm-diameter mycelial plugs of *Diaporthe novem* DJ13 were inoculated into 100 mL of potato dextrose broth (PDB) medium and incubated at 28 °C on a rotary shaker at 200 rpm for 7 days. After incubation, the culture was first filtered through sterile gauze to remove mycelial debris. The resulting filtrate was then sterilized by passage through a 0.22 μm sterile membrane filter (Millipore, Burlington, MA, USA) to obtain a cell-free sterile filtrate. To ensure consistency across all experiments, the fermentation broth was prepared in a single large batch under identical conditions, and aliquots were stored at 4 °C and used within the same experimental series to minimize batch-to-batch variation. The sterile filtrate was used for all subsequent experiments [27].

### 2.3. Effects of the DJ13 Fermentation Broth on C. jiangxiense

Unless otherwise specified, all co-culture experiments were performed as follows: three 5-mm-diameter mycelial plugs of *C. jiangxiense* were inoculated into 150 mL of PDB medium and incubated at 28 °C with shaking at 180 rpm for 5 days. Subsequently, 50 mL of DJ13 fermentation broth was added, and samples were collected at 0, 4, 8, 12, 24, and 48 h after treatment. For the control group, an equal volume (50 mL) of PDB was added instead of the fermentation broth, so that both groups underwent the same dilution (from 150 mL to 200 mL total volume), and any differences could be attributed to the bioactive components in the fermentation broth rather than to dilution effects. All experiments were performed with three independent biological replicates.

#### 2.3.1. Measurement of Electrical Conductivity

Cell membrane permeability was assessed using the electrical conductivity method [28]. At each time point, 5 mL of the culture was centrifuged at 10,000 rpm for 10 min, and the electrical conductivity of the supernatant was measured using a conductivity meter.

#### 2.3.2. Measurement of Extracellular Soluble Sugar Content

**Figure 1:**
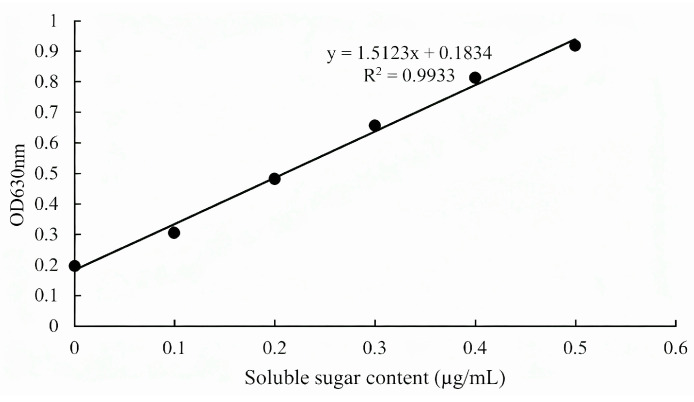
Standard curve of glucose determined by the anthrone colorimetric method at a detection wavelength of 630 nm.

The extracellular soluble sugar content was determined using the anthrone colorimetric method [29,30]. A glucose standard curve was generated by measuring absorbance at 630 nm (Figure 1). For sample measurement, 1 mL of the culture was centrifuged at 10,000 rpm for 10 min, and the supernatant was diluted 20-fold with distilled water. A 0.5 mL aliquot of the diluted solution was mixed with 2 mL of anthrone reagent, incubated in an ice bath for 5 min, heated in a boiling water bath for 10 min, and then cooled. The absorbance was measured at 630 nm, and the soluble sugar content was calculated based on the standard curve.

#### 2.3.3. Determination of Extracellular Macromolecular Substances

The culture was centrifuged at 10,000 rpm for 10 min, and the absorbance of the supernatant was measured at 260 nm (for nucleic acids) and 280 nm (for proteins) to monitor the leakage of intracellular macromolecules.

#### 2.3.4. Determination of Soluble Protein Content

**Figure 2:**
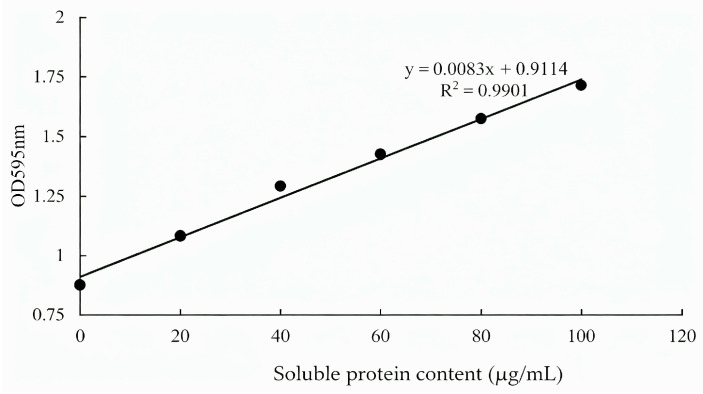
Standard curve of bovine serum albumin (BSA) with detection wavelength at 595 nm.

Soluble protein content was determined according to the method of Fernández-Herrera [31]. A bovine serum albumin (BSA) standard curve was generated by measuring absorbance at 595 nm (Figure 2).

Mycelia were collected, and 0.5 g of mycelia was homogenized on ice in 2 mL of 0.1 mol/L phosphate-buffered saline (PBS, pH 7.5). The homogenate was then adjusted to a final volume of 5 mL with PBS and centrifuged at 10,000 rpm for 20 min at 4 °C. A 1 mL aliquot of the supernatant was mixed with 5 mL of Coomassie Brilliant Blue G-250 staining solution, and after 5 min of incubation, the absorbance was measured at 595 nm [32,33]. The soluble protein content was calculated using the following formula:

(1) $$\mathrm{Protein}\;\mathrm{content}\;(\mu g/g)=\frac{X\times V_{t}}{W\times V_{s}}$$

where *X* is the protein amount (μg) from the BSA standard curve, *V_t_* is the total volume of the extraction solution (mL), *W* is the sample fresh weight (g), and *V_s_* is the volume of the supernatant used for the assay (mL).

#### 2.3.5. Determination of Malondialdehyde (MDA) Content

Mycelia were collected and washed twice with PBS. A 0.5 g aliquot was homogenized in 5 mL of 10% (*w*/*v*) trichloroacetic acid (TCA) [34]. The MDA content was determined using the thiobarbituric acid (TBA) method [35] and calculated as follows:

(2) $$\mathrm{MDA}\;\mathrm{content}\;(\mu \mathrm{mol}/g)=\frac{[6.45\times (OD_{532}-OD_{600})-0.56\times OD_{450}]\times V_{t}}{W}$$

where 6.45 is the millimolar extinction coefficient of the MDA–TBA complex at 532 nm (L·mmol^−1^·cm^−1^), 0.56 is the correction factor for carbohydrate interference, *V_t_* is the total volume of the extraction solution (mL), and *W* is the sample fresh weight (g). These constants are based on the TCA–TBA method and are appropriate for fungal mycelial samples.

#### 2.3.6. Determination of Tricarboxylic Acid (TCA) Cycle Enzyme Activities

Mycelia were collected, and 0.1 g aliquots were used for each enzyme assay. Succinate dehydrogenase (SDH) activity was measured using the SDH assay kit (Solarbio, Beijing, China, BC0955) according to the manufacturer’s instructions, with absorbance recorded at 600 nm. SDH activity (U/g fresh weight) was calculated as follows:

(3) $$\mathrm{SDH}\;\mathrm{activity}=\frac{961.905\times (\Delta A_{\mathrm{sample}}-\Delta A_{\mathrm{blank}})}{W}$$

Malate dehydrogenase (MDH) activity was measured using the NAD-MDH assay kit (Solarbio, BC1075) according to the manufacturer’s instructions, with absorbance recorded at 340 nm. MDH activity (U/g fresh weight) was calculated as follows:

(4) $$\mathrm{MDH}\;\mathrm{activity}=\frac{6430\times (\Delta A_{\mathrm{sample}}-\Delta A_{\mathrm{blank}})}{W}$$

where *W* is the sample fresh weight (g).

#### 2.3.7. Determination of Alkaline Phosphatase (AKP) Activity

**Figure 3:**
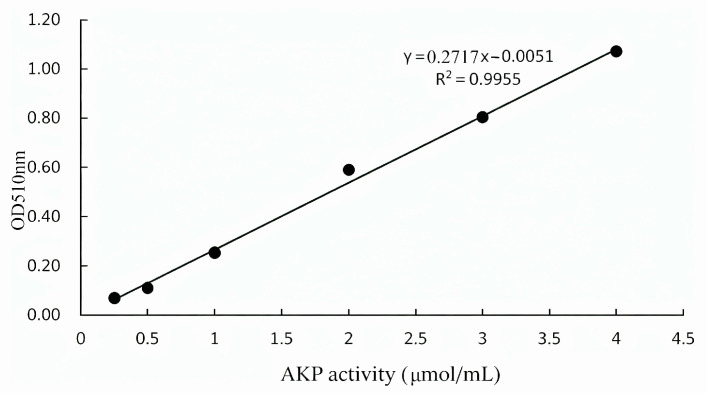
Standard curve of AKP measured at a detection wavelength of 510 nm.

AKP activity was measured using the AKP assay kit (Solarbio, BC2140). A standard curve was generated using the standard solutions provided in the kit (Figure 3). Mycelia (0.1 g) were homogenized in 1 mL of the extraction solution supplied with the kit. The assay was performed according to the manufacturer’s instructions, and the absorbance was recorded at 510 nm. The AKP concentration (x, μmol/mL) was determined by substituting ΔA (A_sample_ − A_control_) into the linear equation of the standard curve.

### 2.4. Transcriptome Analysis

Three 5-mm-diameter mycelial plugs of *C. jiangxiense* were inoculated into 150 mL of Potato Dextrose Broth (PDB) and incubated at 28 °C with shaking at 280 rpm for 3 days. Subsequently, 50 mL of sterile DJ13 fermentation filtrate was added to the treatment group, while an equal volume of PDB was added to the control group. Each treatment group consisted of three independent biological replicates, with each replicate cultured in a separate flask to avoid cross-contamination.

Mycelial morphology was examined every 12 h under a light microscope (Olympus BX53, Olympus Corporation, Tokyo, Japan) to determine the appropriate sampling time point for sequencing. Mycelia were ultimately collected at 48 h post-treatment with the DJ13 fermentation broth. The mycelia were collected by filtration through sterile gauze, rinsed three times with sterile distilled water, thoroughly blotted dry, snap-frozen in liquid nitrogen, and then stored at −80 °C for subsequent use.

Total RNA was extracted from mycelia using TRIzol reagent (Invitrogen, Carlsbad, CA, USA). Library construction and de novo transcriptome sequencing (without a reference genome) were performed by Wuhan Maiwei Biotechnology Co., Ltd. (Wuhan, China). Raw reads were filtered using fastp (v0.23.2) to remove low-quality sequences and obtain high-quality clean reads. The clean reads were then assembled de novo into unigenes using Trinity (v2.15.1). Assembly quality was evaluated by mapping the clean reads back to the assembled unigenes using Bowtie 2 software.

Based on the clean read count matrix, principal component analysis (PCA) and Pearson correlation coefficient analysis were performed to assess inter-sample reproducibility and between-group differences. DEGs were identified using DESeq2 (v1.30.0) with the screening criteria of |log_2_(fold change)| > 2 and a false discovery rate (FDR) < 0.05 after Benjamini–Hochberg correction. GO (Gene Ontology) functional enrichment analysis and KEGG (Kyoto Encyclopedia of Genes and Genomes) pathway enrichment analysis were performed on the DEGs using clusterProfiler (v4.6.0), with a corrected *p*-value < 0.05 considered as the threshold for significant enrichment.

### 2.5. qRT-PCR Validation of RNA-Seq Data

Nine DEGs were randomly selected from the identified DEGs for qRT-PCR validation. Total RNA was extracted from mycelia of the control group (untreated with DJ13 sterile fermentation broth) and the DJ13 fermentation broth-treated group (48 h post-treatment), and reverse-transcribed into cDNA as amplification templates. Glyceraldehyde-3-phosphate dehydrogenase (*GAPDH*) was used as the internal reference gene for normalization.

Total RNA was extracted using TRIzol reagent (Invitrogen, Carlsbad, CA, USA). First-strand cDNA was synthesized using the 5× OneStep RT SuperMix reverse transcription kit (BiologyArk, Shanghai, China, Cat. No. BPC403-01). Gene-specific primers for qRT-PCR were designed using Primer Premier 6 software, and the primer sequences are listed in Supplementary Table S1.

Quantitative PCR amplification was performed using the 2× Plus qPCR MasterMix (BiologyArk, Cat. No. BPC511-01) on a CFX48 ECO™ Real-Time PCR System (Illumina, San Diego, CA, USA). Each 20-μL reaction mixture contained 10 μL of 2× Plus qPCR MasterMix, 0.5 μL each of forward and reverse primers, 100 ng of template cDNA, and nuclease-free water to bring the total volume to 20 μL.

The thermal cycling program was as follows: initial denaturation at 95 °C for 3 min (1 cycle), followed by 40 cycles of 95 °C for 15 s and 60 °C for 1 min (annealing and extension).

All experiments were performed with three independent biological replicates, and each biological replicate included three technical replicates. The relative expression levels of target genes were calculated using the 2^–ΔΔCt^ method. Differences in gene expression between the two groups were analyzed using independent-samples Student’s *t*-test, with *p* < 0.05 considered statistically significant. Statistical analysis and graphing were performed using GraphPad Prism 9.0 and Origin 2024. The correlation between qRT-PCR results and RNA-seq data was evaluated using Pearson’s correlation coefficient.

### 2.6. Statistical Analysis

All physiological and biochemical experiments were performed with three biological replicates. Prior to statistical analysis, normality was assessed using the Shapiro–Wilk test, and homogeneity of variances was evaluated using Levene’s test. For physiological data measured across multiple time points, two-way analysis of variance (ANOVA) was applied with treatment and time as fixed factors, followed by Tukey’s Honestly Significant Difference (HSD) post hoc test for multiple comparisons. For comparisons between two groups at a single time point, an independent samples *t*-test was used. A *p*-value < 0.05 was considered statistically significant, while *p* < 0.01 and *p* < 0.001 were considered highly significant. In the figures, different lowercase letters at the same time point indicate significant differences between groups.

For RNA-seq differential expression analysis, raw count data were analyzed using DESeq2 (v1.30.0). Genes with |log_2_fold change| ≥ 2 and a false discovery rate (FDR) < 0.05 (Benjamini–Hochberg correction) were defined as DEGs. Correlation analysis between RNA-seq and qRT-PCR data was performed using Pearson’s correlation coefficient.

All statistical analyses were conducted using SPSS (version 27.0), and figures were generated using GraphPad Prism (version 9.0) and Origin 2024.

## 3. Results

### 3.1. Effects of the DJ13 Fermentation Broth on C. jiangxiense

#### 3.1.1. Effect on Electrical Conductivity of *C. jiangxiense*

**Figure 4:**
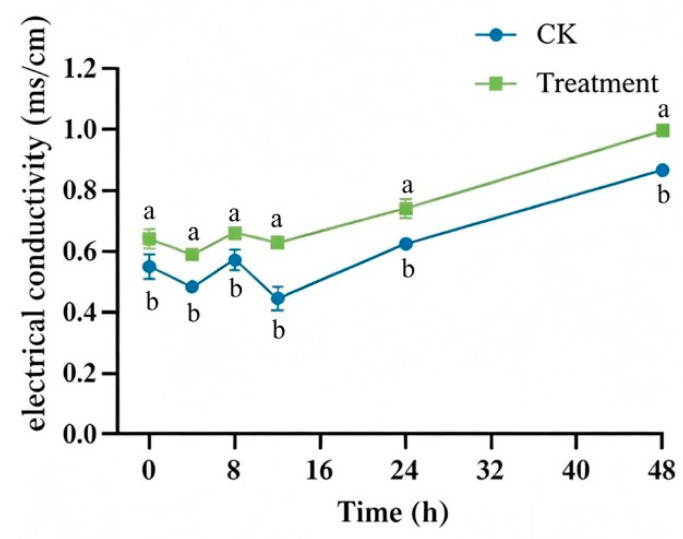
Effects of DJ13 fermentation broth on the electrical conductivity of *C. jiangxiense*. CK represents the control group, and Treatment represents the group treated with DJ13 fermentation broth. Different lowercase letters indicate significant differences between the two groups at the same time point (*p* < 0.05). Error bars represent the standard error of biological replicates.

As shown in Figure 4, the relative electrical conductivity of *C. jiangxiense* increased over time after treatment with DJ13 fermentation broth. At 12 h after treatment, the conductivity was 1.41-fold higher than that in the control group and remained significantly elevated throughout the observation period. These results indicate that DJ13 fermentation broth compromised pathogen cell integrity, as reflected by increased electrolyte leakage and electrical conductivity.

#### 3.1.2. Effect on Soluble Sugar Content of *C. jiangxiense*

**Figure 5:**
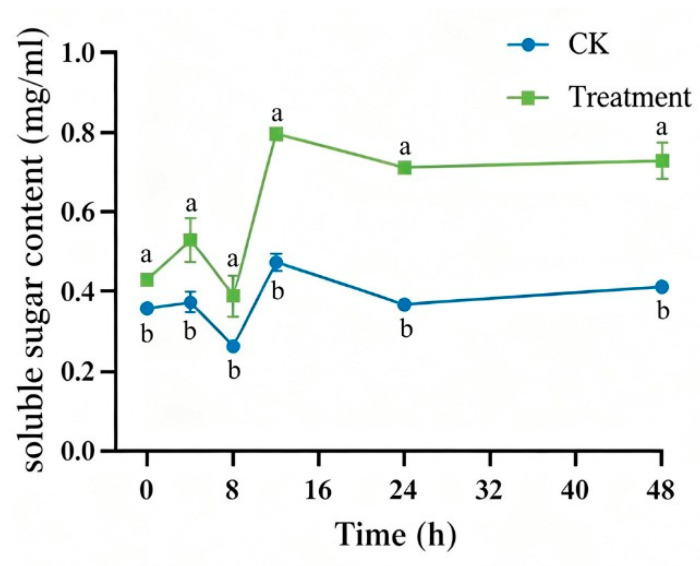
Effects of DJ13 fermentation broth treatment on extracellular soluble sugar content of *C. jiangxiense*. CK represents the control group, and Treatment represents the group treated with DJ13 fermentation broth. Different lowercase letters indicate significant differences between the two groups at the same time point (*p* < 0.05). Error bars represent the standard error of biological replicates.

A calibration curve was established for soluble sugar quantification (y = 1.5123x + 0.1834, R^2^ = 0.9933; Figure 1). The curve showed good linearity and was suitable for subsequent analysis. Soluble sugar levels fluctuated over time in both the control and DJ13-treated groups. However, levels in the control group were consistently lower than those in the treatment group. In the DJ13-treated group, soluble sugar content reached a maximum of 0.89 mg/mL at 12 h, which was 1.90-fold higher than that in the control group (Figure 5). These results suggest that DJ13 fermentation broth disrupted the cell membrane structure of *C. jiangxiense*, leading to increased membrane permeability and leakage of intracellular sugars into the extracellular medium.

#### 3.1.3. Effect on Extracellular Macromolecular Substances in *C. jiangxiense*

**Figure 6:**
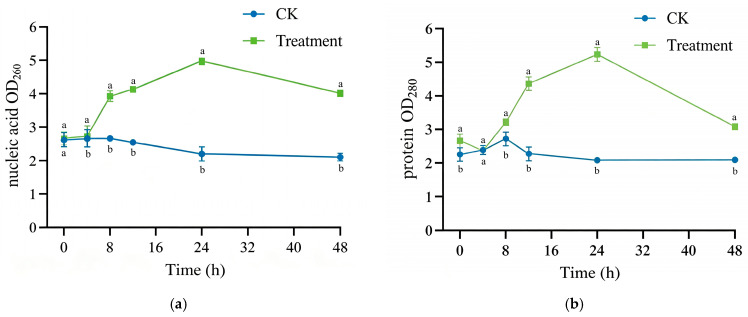
Effects of DJ13 fermentation broth on the leakage of extracellular nucleic acids and proteins in *C. jiangxiense*. (**a**) Nucleic acid absorbance at 260 nm (OD_260_); (**b**) protein absorbance at 280 nm (OD_280_). CK represents the control group, and Treatment represents the group treated with DJ13 fermentation broth. Different lowercase letters indicate significant differences between the two groups at the same time point (*p* < 0.05). Error bars represent the standard error of biological replicates.

Cell membrane damage can cause leakage of intracellular inorganic ions and release of macromolecular substances, including nucleic acids and proteins, into the extracellular environment. Therefore, absorbance at 260 nm and 280 nm was measured to assess the leakage of nucleic acids and proteins, respectively, after treatment with DJ13 fermentation broth. As shown in Figure 6, the control and treatment groups showed distinct patterns. In the control group, absorbance values for nucleic acids and proteins increased slightly during the first 8 h, then declined continuously after 8 h. After 24 h, both values stabilized and remained at a relatively low level of approximately 2.1. In contrast, absorbance values in *C. jiangxiense* treated with DJ13 fermentation broth increased rapidly and continuously from 0 to 24 h, reaching peaks of approximately 5.09 for nucleic acids and 5.22 for proteins at 24 h. Although these values decreased thereafter, they remained significantly higher than those in the control group at 48 h. These findings suggest that DJ13 fermentation broth affected nucleic acid and protein metabolism in *C. jiangxiense*, possibly by disrupting cellular structures and interfering with macromolecular metabolism.

#### 3.1.4. Effect on Soluble Protein Content of *C. jiangxiense*

**Figure 7:**
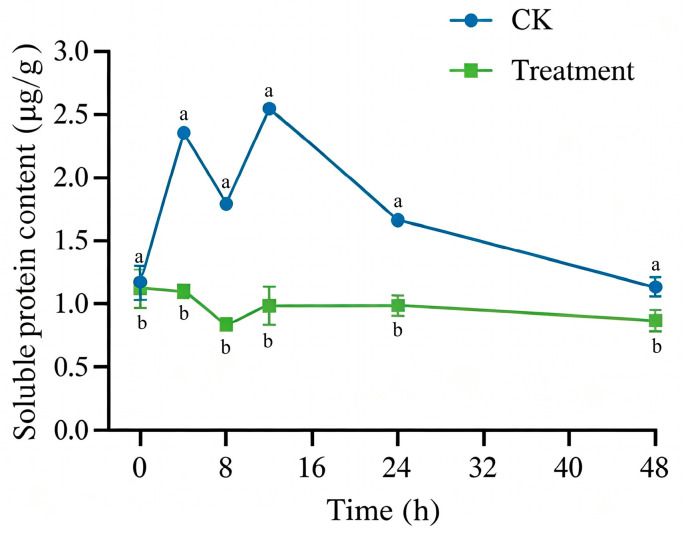
Effects of DJ13 fermentation broth treatment on intracellular soluble protein content of *C. jiangxiense*. CK represents the control group, and Treatment represents the group treated with DJ13 fermentation broth. Different lowercase letters indicate significant differences between the two groups at the same time point (*p* < 0.05). Error bars represent the standard error of biological replicates.

A calibration curve was established using bovine serum albumin (BSA) as the standard (y = 0.0083x + 0.9114, R^2^ = 0.9901; Figure 2). The curve showed good linearity and was suitable for soluble protein quantification. As shown in Figure 7, soluble protein content fluctuated over time in both the control and DJ13-treated groups. However, levels in the treatment group remained consistently lower than those in the control group. At 12 h, the soluble protein content in the treatment group was 0.99 μg/g, compared with 2.54 μg/g in the control group, representing a 61.02% reduction. These results suggest that DJ13 fermentation broth may interfere with protein synthesis in *C. jiangxiense*, thereby disrupting normal physiological activity and potentially contributing to cell death.

#### 3.1.5. Effect on Malondialdehyde (MDA) Content of *C. jiangxiense*

**Figure 8:**
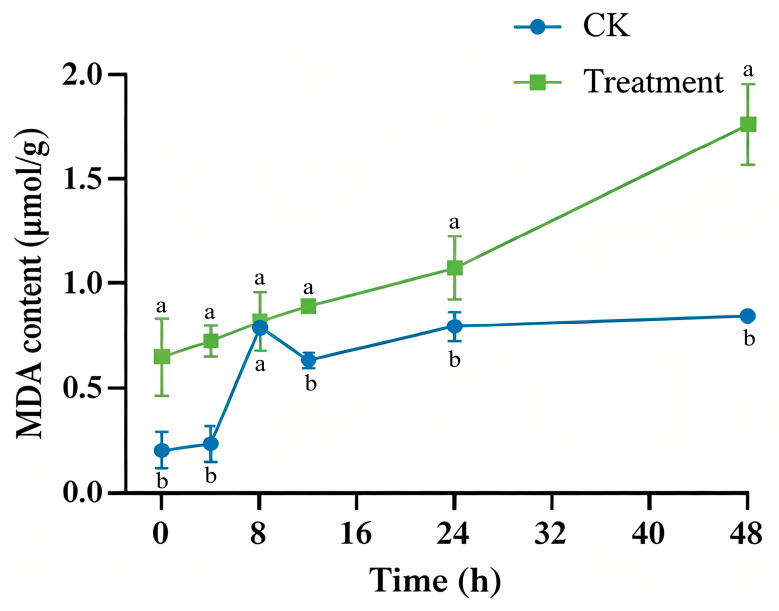
Effects of DJ13 fermentation broth on malondialdehyde (MDA) content in *C. jiangxiense*. CK represents the control group, and Treatment represents the group treated with DJ13 fermentation broth. Different lowercase letters indicate significant differences between the two groups at the same time point (*p* < 0.05). Error bars represent the standard error of biological replicates.

MDA is an important indicator of membrane lipid peroxidation, and its content reflects the extent of cell membrane damage. As shown in Figure 8, MDA content in *C. jiangxiense* increased sharply after treatment with DJ13 fermentation broth and reached a maximum of 0.84 μmol/g at 48 h. In the control group, MDA content increased rapidly only during the first 8 h, after which the rate of increase slowed markedly. Throughout the observation period, MDA levels in the control group remained lower than those in the treatment group.

#### 3.1.6. Effects on the Activities of TCA Cycle Enzymes in *C. jiangxiense*

**Figure 9:**
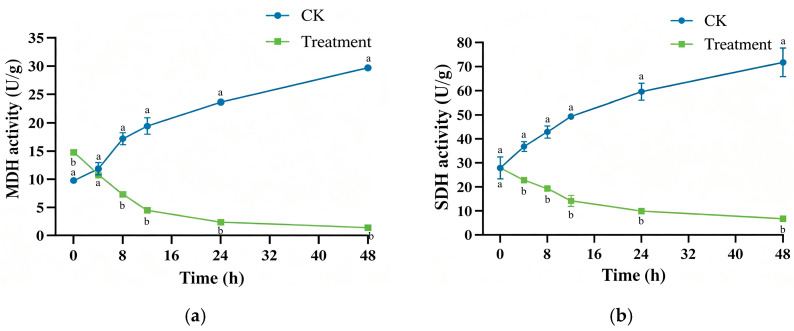
Effects of DJ13 fermentation broth on the activities of key enzymes in the tricarboxylic acid (TCA) cycle in *C. jiangxiense*. (**a**) Malate dehydrogenase (MDH) activity; (**b**) succinate dehydrogenase (SDH) activity. CK represents the control group, and Treatment represents the group treated with DJ13 fermentation broth. Different lowercase letters indicate significant differences between the two groups at the same time point (*p* < 0.05). Error bars represent the standard error of biological replicates.

Malate dehydrogenase (MDH) and succinate dehydrogenase (SDH) are key regulatory enzymes in cellular energy metabolism, and their activities reflect cellular energy status. As shown in Figure 9, both MDH and SDH activities in *C. jiangxiense* mycelia decreased sharply within the first 4 h after treatment with DJ13 fermentation broth. MDH activity declined from 14.73 U/g at 0 h to 2.35 U/g at 24 h, after which the decrease slowed. SDH activity decreased from 27.82 U/g at 0 h to 9.86 U/g at 24 h and further declined to 6.79 U/g at 48 h. In contrast, MDH and SDH activities in the control group increased gradually over time. These results indicate that DJ13 fermentation broth markedly inhibited MDH and SDH activities in the pathogen, suggesting disruption of the TCA cycle and impairment of cellular energy metabolism.

#### 3.1.7. Effect on AKP Activity of *C. jiangxiense*

**Figure 10:**
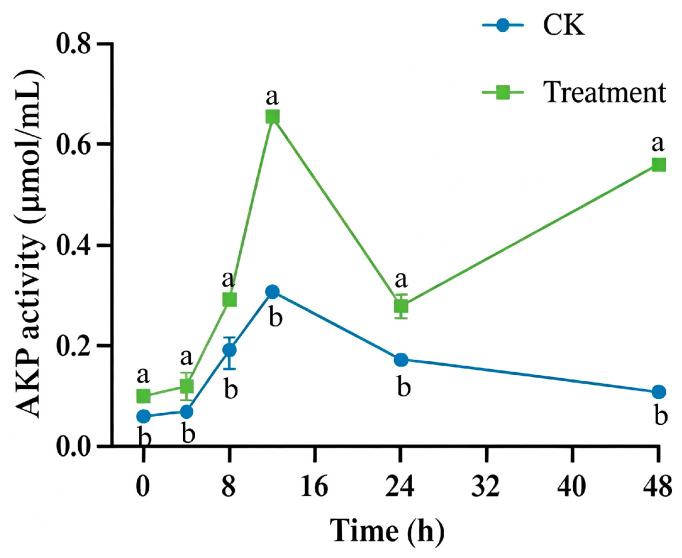
Effects of DJ13 fermentation broth on extracellular AKP activity in *C. jiangxiense*. CK represents the control group, and Treatment represents the group treated with DJ13 fermentation broth. Different lowercase letters indicate significant differences between the two groups at the same time point (*p* < 0.05). Error bars represent the standard error of biological replicates.

Under normal physiological conditions, AKP is localized between the cell membrane and the cell wall. When the cell wall is damaged, AKP is released into the surrounding medium, resulting in increased extracellular AKP activity. In this study, AKP activity was measured to assess cell wall integrity in *C. jiangxiense* after treatment with DJ13 fermentation broth. As shown in Figure 10, AKP activity increased rapidly from 0 to 12 h after treatment and reached a peak of 0.66 μmol/mL at 12 h. Although AKP activity decreased at 24 h, it remained higher than that in the control group at 48 h. In the control group, AKP activity increased modestly from 0 to 12 h, peaked at 0.31 μmol/mL at 12 h, and then declined continuously. These results suggest that DJ13 fermentation broth disrupted the cell wall structure of *C. jiangxiense*, which may be one mechanism underlying its antifungal activity.

### 3.2. Effect of Diaporthe novem DJ13 on the Spore Morphology of C. jiangxiense

**Figure 11:**
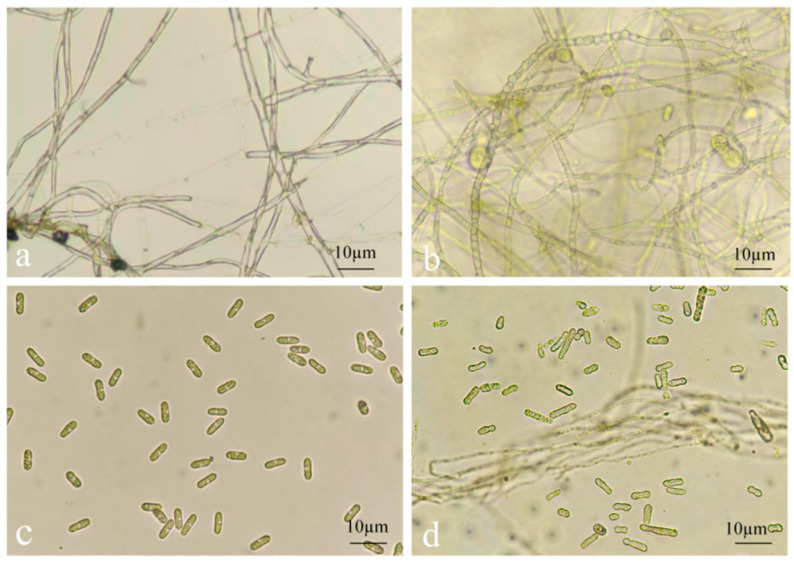
Morphology of mycelia and spores of *C. jiangxiense* co-cultured with DJ13 fermentation broth for 48 h. (**a**) Untreated mycelia; (**b**) Treated mycelia; (**c**) Untreated spores; (**d**) Treated spores.

Figure 11 shows the morphological changes in the mycelia and spores of *C. jiangxiense* during co-culture with DJ13, observed at 12-h intervals. After 48 h of co-culture, *C. jiangxiense* mycelia showed surface wrinkling, aggregation, hyphal swelling, and nodular structures (Figure 11b), whereas the spores showed distortion and lysis (Figure 11d). In contrast, mycelia and spores in the control group retained normal morphology at 48 h (Figure 11a,c). Based on these observations, mycelia from the 48-h co-culture were collected for subsequent analysis. Three biological replicates were included in each group, yielding six mycelial samples in total.

### 3.3. Transcriptome Analysis of C. jiangxiense in Response to DJ13 Fermentation Broth Treatment

#### 3.3.1. RNA-Seq Data Quality Assessment and Inter-Sample Relationship Analysis

**Figure 12:**
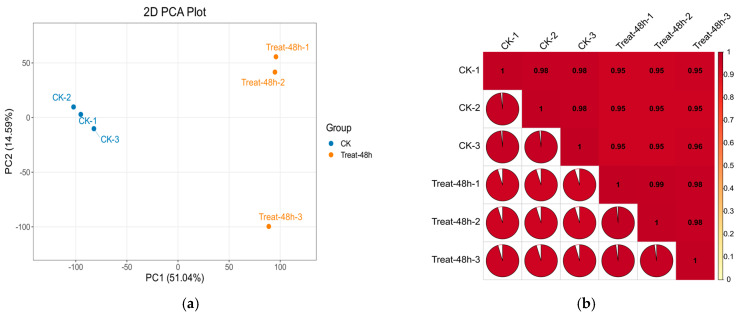
Assessment of reproducibility and intergroup differences in transcriptomic expression profiles. (**a**) Two-dimensional principal component analysis (PCA) plot. PC1 and PC2 explained 51.04% and 14.58% of the total variance, respectively. CK represents the control group, and Treat-48h represents the group treated with DJ13 fermentation broth for 48 h. (**b**) Heatmap of Pearson correlation coefficients among samples. Values in each block indicate the correlation coefficient between paired samples.

Sequencing generated 49.73–61.02 million raw reads per sample. After quality filtering, 48.35–58.61 million clean reads were retained. The percentages of Q20 and Q30 bases exceeded 98.88% and 94.99%, respectively, indicating high sequencing accuracy (Table 1). Principal component analysis (PCA) showed that PC1 and PC2 together explained 65.63% of the total variance (Figure 12a). PC1 accounted for 51.04% of the variance and clearly separated the control group (CK) from the 48-h treatment group (Treat-48h), indicating that DJ13 fermentation broth markedly altered the global transcriptional profile of *C. jiangxiense*. The inter-sample correlation heatmap is shown in Figure 12b. Pearson correlation coefficients among biological replicates within each group were all greater than 0.95, demonstrating high reproducibility. These results indicate that the transcriptomic data were reliable and suitable for downstream analysis.

#### 3.3.2. Identification of DEGs

**Figure 13:**
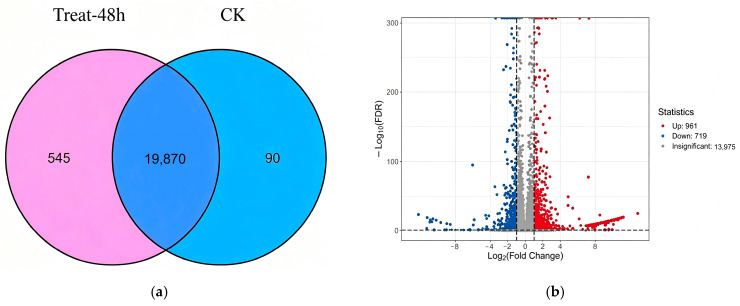
Gene distribution and DEG statistics between the CK and Treat-48h groups. (**a**) Venn diagram of genes detected in the two groups. The overlapping region represents shared genes, whereas the independent regions on the left and right represent genes unique to Treat-48h and CK, respectively. (**b**) Volcano plot of DEGs. The x-axis indicates log_2_(fold change), and the y-axis indicates −log_10_(FDR). Red dots denote significantly up-regulated genes, blue dots denote significantly down-regulated genes, and gray dots represent genes without significant expression differences. CK represents the control group, and Treat-48h represents the group treated with DJ13 fermentation broth for 48 h.

Differential expression analysis was performed using untreated *C. jiangxiense* mycelia as the control group (CK) and mycelia treated with 25% DJ13 fermentation broth for 48 h as the treatment group (Treat-48h). DEGs were identified using thresholds of |log_2_ fold change| ≥ 2 and FDR < 0.05. Venn diagram analysis detected 21,405 genes across the two groups, of which 19,870 were shared, accounting for 92.8% of all detected genes (Figure 13a). This result indicates that the core transcript repertoire was largely conserved between the two groups. Among group-specific genes, 545 were detected only in the Treat-48h group, whereas 90 were detected only in the CK group, suggesting that 48-h treatment induced the expression of many treatment-specific genes.

The volcano plot shows the overall distribution and statistical significance of the DEGs (Figure 13b). A total of 1680 DEGs were identified, including 961 significantly up-regulated genes and 719 significantly down-regulated genes. Up-regulated genes outnumbered down-regulated genes, and some genes showed an absolute log_2_ fold change greater than 8. These findings indicate that DJ13 fermentation broth exerted a strong regulatory effect on pathogen gene expression. They are also consistent with the clear separation along PC1 in the PCA plot (Figure 12a), further supporting that the 48-h treatment substantially altered the biological state of *C. jiangxiense* by modulating the expression of numerous functional genes.

#### 3.3.3. GO Enrichment Analysis of DEGs

**Figure 14:**
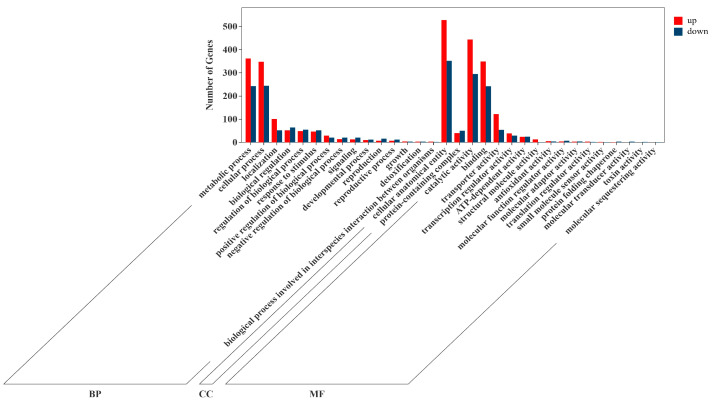
Level 2 GO enrichment classification of DEGs in *C. jiangxiense* treated with the biocontrol fungus *Diaporthe novem*.

GO enrichment analysis of the CK vs. Treat-48h comparison is shown in Figure 14. In the Biological Process category, DEGs were significantly enriched in metabolic process, cellular process, localization, and biological regulation. In the Cellular Component category, DEGs were significantly enriched in cellular anatomical entities and protein-containing complexes. In the Molecular Function category, DEGs were significantly enriched in catalytic activity, binding, transporter activity, transcription regulator activity, and ATP-dependent activity.

#### 3.3.4. KEGG Enrichment Analysis of DEGs

**Figure 15:**
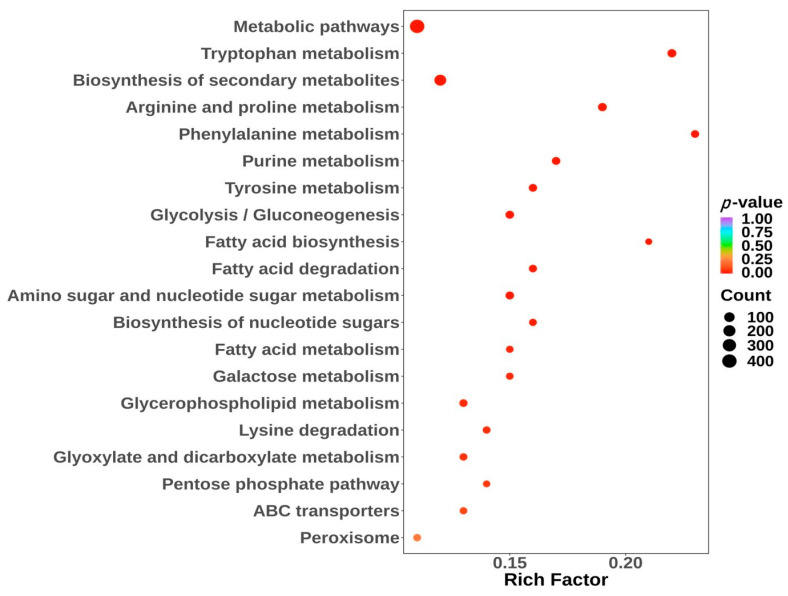
Significantly enriched KEGG pathways of DEGs in *C. jiangxiense* treated with the biocontrol fungus *Diaporthe novem* DJ13.

A total of 1828 DEGs between the CK and Treat-48h groups were annotated to 130 KEGG pathways. Figure 15 shows the 20 most significantly enriched pathways. These pathways mainly included metabolic pathways, biosynthesis of secondary metabolites, tryptophan metabolism, arginine and proline metabolism, glycolysis, glycerophospholipid metabolism, ABC transporters, and peroxisomes. Most of these pathways are associated with growth and metabolism, cell structure synthesis, and adaptation to environmental stress. These results suggest that DJ13 fermentation broth may inhibit the growth of *C. jiangxiense* through coordinated regulation of metabolic pathways, secondary metabolite biosynthesis, and ABC transporter activity.

#### 3.3.5. Analysis of DEGs in *Colletotrichum jiangxiense*

##### Effects of DJ13 Fermentation Broth on Cell-Membrane-Synthesis-Related Genes in *C. jiangxiense*

ATP-binding cassette (ABC) transporters and the major facilitator superfamily (MFS) are two major families of membrane-localized transmembrane transporters. Several genes encoding efflux pumps in these families were significantly up-regulated after DJ13 fermentation broth treatment. For example, the ABC transporter gene Cluster-12281.2 (*atnG-7*) was up-regulated, suggesting that *C. jiangxiense* may enhance efflux activity to reduce the intracellular accumulation of antimicrobial compounds. However, the functional contribution of these transporters requires further validation. Similarly, the MFS transporter gene Cluster-5639.0 (*CGCA056_v014182*) was significantly up-regulated, which may contribute to substrate efflux or regulation of membrane stability, although its specific role remains speculative.

In contrast, several genes involved in membrane structure and metabolism were suppressed. For instance, Cluster-14541.6 (*CGCS363_v008146*), annotated as a monoacylglycerol lipase involved in membrane lipid hydrolysis, was almost completely inhibited. Its down-regulation may help limit further damage to membrane structures, although this interpretation remains hypothetical. Additionally, Cluster-5171.0 (*KHT2-1*) and Cluster-6458.0 (*CGCA056_v004114*) were down-regulated. These genes show high homology to a hexose transporter and an integral membrane protein, respectively. Under conditions of membrane potential disruption, *C. jiangxiense* may reduce nutrient uptake to lower the risk of osmotic imbalance.

###### Effect of DJ13 Fermentation Broth on Cell-Wall-Related Genes in *C. jiangxiense*

**Figure 16:**
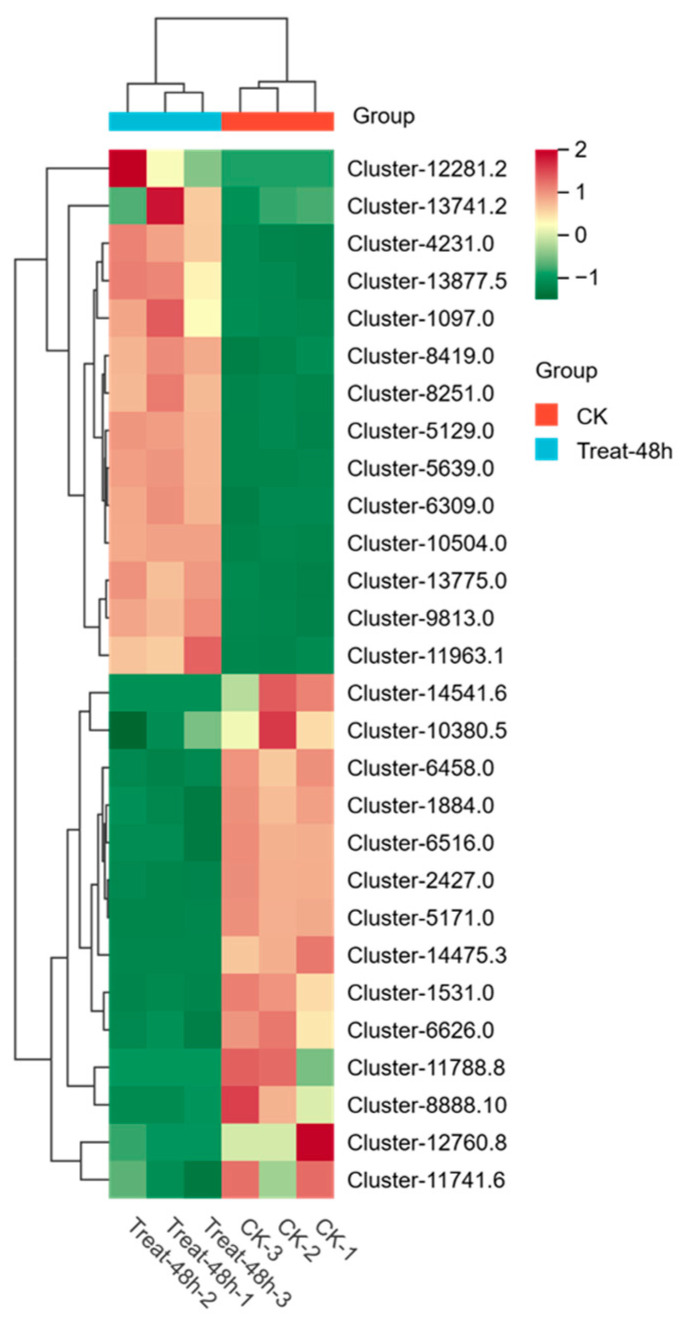
Hierarchical clustering heatmap of DEGs after 48 h of co-culture. Rows represent individual DEG clusters, and columns represent individual samples. The color gradient indicates normalized gene expression levels, with red representing high expression and green representing low expression. CK indicates the untreated control group, and Treat-48h indicates the group treated with DJ13 fermentation broth for 48 h.

Transcriptomic analysis showed that DJ13 fermentation broth markedly altered the expression of multiple genes involved in cell wall metabolism in *C. jiangxiense* (Figure 16). Genes responsible for the degradation of major cell wall components were significantly up-regulated. These included chitinase Cluster-13877.5 (*CHI1-1*), polysaccharide monooxygenase Cluster-4231.0 (*Cel61a-2*), and β-glucosidase Cluster-8888.13 (*GCG54_00008211*). Conversely, genes associated with cell wall synthesis and remodeling were significantly down-regulated, including α-glucanase Cluster-6626.0 (Mutanase) and the oligosaccharide-processing enzyme Cluster-14475.3 (*CDEST_06436*). These findings suggest that DJ13 treatment may simultaneously promote cell wall degradation and inhibit cell wall synthesis in the pathogen. Direct biochemical validation is needed to confirm these effects.

####### Effect of DJ13 Fermentation Broth on Antioxidant-Stress-Related Genes in *C. jiangxiense*

Transcriptomic analysis indicated that DJ13 fermentation broth significantly affected the expression of multiple genes associated with antioxidant defense and damage repair in *C. jiangxiense* (Figure 16). Two genes encoding DASH family cryptochrome/DNA photolyases, Cluster-2427.0 (*CGCA056_v012201*) and Cluster-6516.0 (*CDEST_11904*), were significantly down-regulated. In contrast, genes involved in purine metabolism and peroxisome-associated oxidation were significantly up-regulated, including xanthine dehydrogenase Cluster-9813.0 (*GCG54_00009806*) and L-pipecolic acid oxidase Cluster-13775.0 (*fap2-4*). These results suggest that DJ13 treatment may alter the intracellular redox status of the pathogen, which may be associated with the observed transcriptional reprogramming.

######## Effects of DJ13 Fermentation Broth on Respiration-and Energy-Metabolism-Related Genes in *C. jiangxiense*

Transcriptomic analysis showed that DJ13 fermentation broth substantially affected energy metabolism pathways in *C. jiangxiense* (Figure 16). Multiple genes encoding FAD-dependent oxidoreductases, including Cluster-10504.0 (*CkaCkLH20_13113*) and Cluster-8251.0 (*OpS4-7*), were significantly up-regulated. In contrast, genes involved in respiratory substrate metabolism were generally down-regulated, including trimethyllysine dioxygenase Cluster-12760.8 (*CBS-1*) and peroxisomal CoA synthetase Cluster-11788.8 (*CGGC5_v006464*). Together, these changes suggest that DJ13 fermentation broth may affect pathogen respiration at multiple levels. The detailed annotations of each gene are shown in Table 2.

### 3.4. qRT-PCR Validation of DEGs

**Figure 17:**
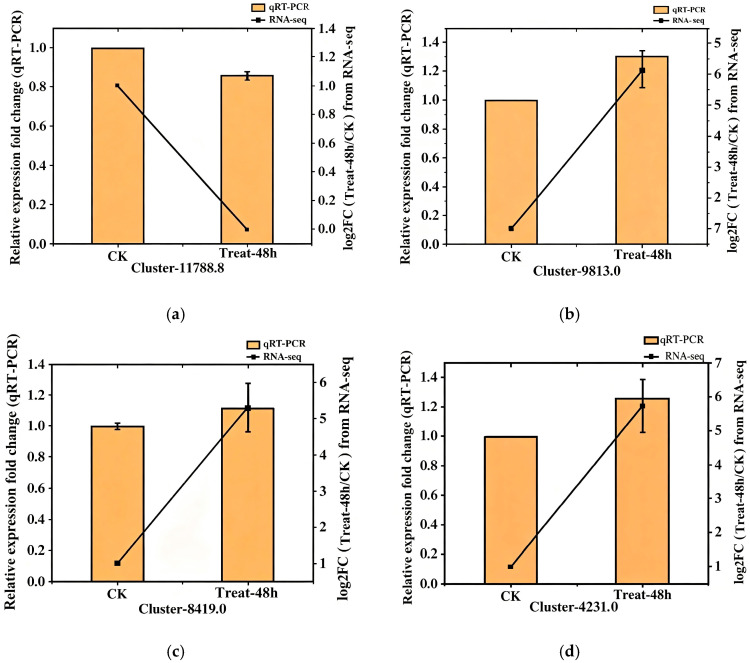

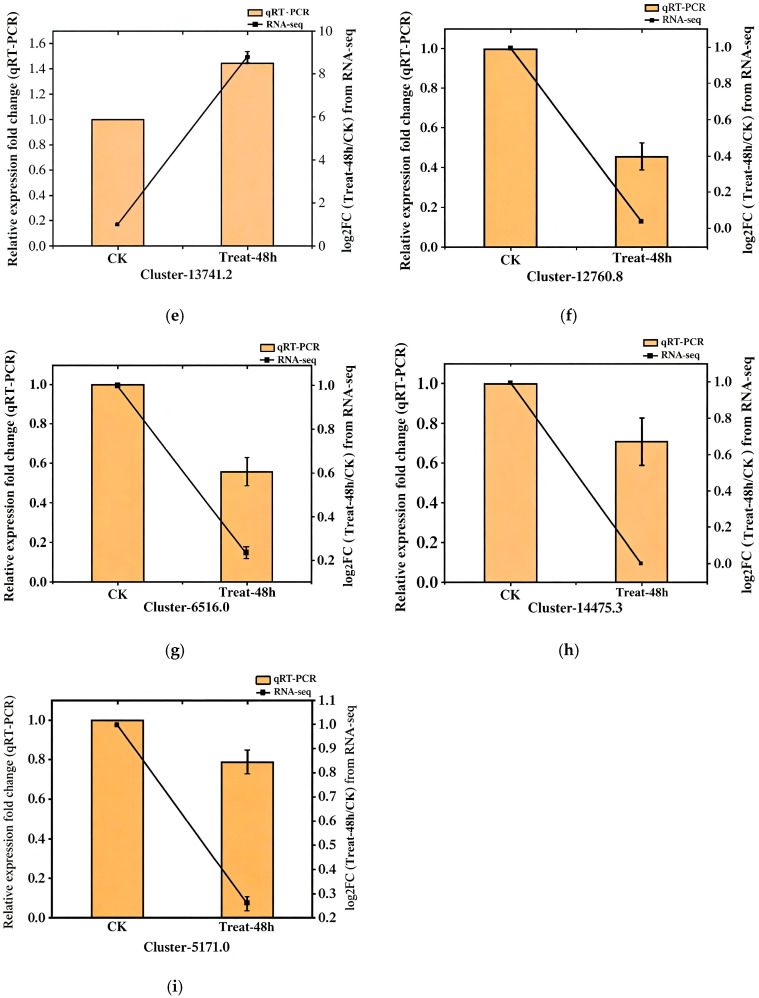
Validation of the consistency between RNA-seq and qRT-PCR expression trends for nine DEGs. (**a**) Cluster-11788.8; (**b**) Cluster-9813.0; (**c**) Cluster-8419.0; (**d**) Cluster-4231.0; (**e**) Cluster-13741.2; (**f**) Cluster-12760.8; (**g**) Cluster-6516.0; (**h**) Cluster-14475.3; (**i**) Cluster-5171.0. Orange bars represent the relative gene expression fold changes measured by qRT-PCR (left y-axis), with expression in the CK group normalized to 1. The black line represents the log_2_(Treat-48h/CK) values obtained from the transcriptomic data (right y-axis). The scale of the right y-axis for log_2_FC in each subplot was adjusted according to the magnitude of expression differences for each gene. CK indicates the untreated control group, and Treat-48h indicates the group treated with DJ13 fermentation broth for 48 h. Error bars represent the standard errors of biological replicates. Expression differences between the two groups were analyzed using an independent-samples Student’s *t*-test.

To validate the accuracy of the transcriptomic sequencing results, nine representative unigenes were selected from the DEGs for qRT-PCR analysis. These included four up-regulated genes (Cluster-9813.0, Cluster-8419.0, Cluster-4231.0, and Cluster-13741.2) and five down-regulated genes (Cluster-12760.8, Cluster-6516.0, Cluster-14475.3, Cluster-5171.0, and Cluster-11788.8) after treatment (Figure 17).

Using *GAPDH* as the internal reference gene, the relative expression level of each gene in the CK group and the DJ13 fermentation broth-treated group at 48 h was calculated using the 2^–ΔΔCt^ method. The transcriptomic log_2_ fold change was defined as log_2_(Treat-48h/CK).

The qRT-PCR results showed that the expression trends of all nine genes were consistent with the RNA-seq data. The up-regulated genes had significantly higher relative expression levels in the Treat-48h group than in the CK group, corresponding to higher log_2_(Treat-48h/CK) values in the transcriptomic data. Conversely, the down-regulated genes showed lower expression levels in the Treat-48h group than in the CK group, corresponding to lower log_2_(Treat-48h/CK) values.

Pearson correlation analysis of the two quantitative datasets showed strong concordance between qRT-PCR and RNA-seq results. These findings confirm the reliability of the transcriptomic sequencing data and support their use in subsequent functional enrichment analyses.

## 4. Discussion

The antifungal activity of microbial fermentation broths is commonly attributed to their ability to disrupt the structural integrity of pathogen cells. In this study, physiological assays and transcriptomic analysis were integrated to investigate the mechanism by which *D. novem* DJ13 fermentation broth inhibits *C. jiangxiense*. The combined evidence suggests that DJ13 exerts its antifungal activity through coordinated effects on the cell membrane and cell wall, accompanied by oxidative stress and disrupted energy metabolism.

The earliest and most pronounced physiological responses to DJ13 treatment were increased membrane permeability and leakage of intracellular contents. Electrical conductivity, soluble sugar leakage, and the efflux of nucleic acids and proteins all increased continuously during the 48-h observation period (Figure 4, Figure 5 and Figure 6). These findings are consistent with the reported modes of action of other biocontrol fermentation products, including *Paenibacillus* sp. NK3-4 against *Exserohilum turcicum* and *Bacillus altitudinis* GS-16 against *Colletotrichum gloeosporioides* [33,36], in which membrane destabilization is a primary event. In the present system, these permeability changes occurred rapidly, within 12 h of treatment, suggesting that membrane damage represents an early target of DJ13 fermentation broth rather than a late consequence of general cellular deterioration.

Membrane damage was further supported by the significant accumulation of MDA, a well-established marker of ROS-mediated lipid peroxidation (Figure 8) [37]. Although MDA is an indirect indicator, its sustained increase during treatment strongly suggests that ROS production amplified the disruption of the phospholipid bilayer. ROS accumulation may have been directly induced by metabolites in the fermentation broth or may have occurred secondarily to membrane dysfunction. This pattern is consistent with the findings of Wang et al. [38], who reported parallel increases in MDA content and electrical conductivity in *Fusarium solani* treated with an endophytic fungal filtrate. In the present study, however, MDA remained elevated beyond 24 h rather than showing a transient peak. This finding indicates that oxidative damage was not limited to an early stress response but persisted as a sustained component of the antifungal effect.

At the transcriptomic level, the expression patterns of membrane-related genes provided molecular support for these physiological observations. ABC transporter genes, which encode efflux pumps involved in the active removal of xenobiotics, were significantly up-regulated, including Cluster-12281.2 and related genes. This up-regulation may reflect a defensive response, but its exact functional contribution to the antifungal effect requires further validation. Conversely, genes encoding structural or metabolic components of the membrane, including monoacylglycerol lipase and integral membrane proteins, were significantly down-regulated. This pattern suggests a reduced capacity for membrane repair or maintenance. The concurrent up-regulation of efflux pump genes and down-regulation of membrane structural genes is consistent with transcriptomic responses reported in other pathogenic fungi treated with biocontrol agents [21,39]. These findings support the interpretation that membrane perturbation is a major and sustained effect of DJ13 treatment.

In addition to the membrane, the cell wall also appeared to be compromised by DJ13 treatment. AKP is localized between the cell membrane and cell wall, and its activity can reflect cell wall integrity [40]. Changes in AKP activity have also been associated with fungal cell wall damage and reduced virulence [41]. In this study, AKP activity was significantly elevated in the extracellular medium (Figure 10), indicating disruption of the cell wall barrier. This interpretation was further supported by the transcriptomic data. Genes encoding cell-wall-degrading enzymes, including chitinase (Cluster-13877.5), polysaccharide monooxygenase (Cluster-4231.0), and β-glucosidase (Cluster-8888.13), were significantly up-regulated, whereas genes associated with cell wall synthesis, such as α-glucanase (Cluster-6626.0), were down-regulated. These findings are consistent with previous evidence that fermentation products of *Chaetomium globosum* synergistically inhibit *Phytophthora capsici* through cell wall degradation and cell membrane disruption [42]. However, the observed transcriptional changes alone do not demonstrate that DJ13 directly degrades the pathogen’s cell wall; they only indicate a correlation. The similar temporal patterns of increased membrane permeability and AKP leakage suggest that the cell membrane and cell wall were affected in parallel, possibly by different classes of bioactive metabolites present in the crude fermentation broth.

While membrane and cell wall disruption represent major forms of structural damage, the antifungal activity of DJ13 also appeared to interfere with core metabolic processes. The activities of two key tricarboxylic acid (TCA) cycle enzymes, malate dehydrogenase (MDH) and succinate dehydrogenase (SDH), declined sharply within 4 h of treatment and remained suppressed throughout the experimental period (Figure 9). Given that MDH and SDH are essential enzymes that sustain TCA cycle flux, their inhibition would be expected to reduce ATP production and impair energy-dependent cellular functions, including membrane repair and macromolecular synthesis [43,44]. Suppression of MDH and SDH occurred earlier than maximal membrane leakage, which peaked at 24–48 h. This temporal difference suggests that energy depletion was not merely a downstream consequence of membrane damage but may have represented an independent early event. This distinction is important because it implies that DJ13 fermentation broth may contain metabolites that directly target respiratory enzymes in addition to metabolites that disrupt membrane structures. A similar dual effect on membrane integrity and TCA cycle enzyme activities was reported by Zhang et al. [45] for *Bacillus velezensis* extracts, suggesting that multi-target activity may be a common feature of effective biocontrol agents.

At the transcriptomic level, genes involved in respiratory substrate metabolism, including trimethyllysine dioxygenase (Cluster-12760.8) and peroxisomal CoA synthetase (Cluster-11788.8), generally showed reduced expression. This pattern was consistent with the decreased MDH and SDH activities. Conversely, FAD-dependent oxidoreductase genes, including Cluster-10504.0 and Cluster-8251.0, were up-regulated. This apparent discrepancy may indicate that the pathogen attempted to reroute electron flow through alternative oxidation pathways when the canonical TCA cycle was impaired. It may also reflect a stress response to the accumulation of reducing equivalents. Regardless of the underlying mechanism, the combined physiological and transcriptomic evidence indicates a state of metabolic imbalance, in which energy production is constrained while the demand for cellular repair remains high. This imbalance would further accelerate cellular deterioration.

Alongside the disruption of energy metabolism, oxidative stress appeared to act as both an amplifier and a consequence of the antifungal effect. Although ROS levels were not directly measured, the persistent increase in MDA content strongly suggests sustained oxidative damage [37]. Transcriptomic analysis showed down-regulation of DASH family cryptochrome genes, including Cluster-2427.0 and Cluster-6516.0, which encode DNA repair enzymes. In contrast, ROS generation-associated genes, including xanthine dehydrogenase (Cluster-9813.0) and L-pipecolic acid oxidase (Cluster-13775.0), were up-regulated. This transcriptional pattern is consistent with the observations of Park et al. [46] in endophyte-treated pathogenic fungi, in which ROS accumulation was accompanied by repression of antioxidant repair systems. Nevertheless, without direct ROS measurement, this remains an inference based on gene expression trends. The simultaneous increase in ROS-related gene expression and reduction in DNA repair capacity may create a self-reinforcing cycle. In this process, membrane lipid peroxidation promotes further ROS accumulation, which then causes additional membrane damage and ultimately overwhelms the defensive capacity of the pathogen.

Several methodological limitations should be acknowledged. First, all experiments were conducted in vitro and were not validated in living rhododendron plants. This experimental system was useful for excluding host- and environment-related variables and for defining direct physiological and molecular responses. However, the actual biocontrol efficacy of DJ13 in planta, its potential phytotoxicity, and its compatibility with the rhododendron microbiome remain to be evaluated. Moreover, it should be emphasized that the present study exclusively used cell-free sterile fermentation filtrate of DJ13. The observed antifungal effects can therefore only be attributed to the extracellular metabolites secreted into the broth, and the proposed model describes the action of the fermentation broth rather than the living endophytic fungus. The biocontrol mechanisms of living *Diaporthe novem* in planta may additionally involve mycoparasitism, competition, and induced host resistance, which cannot be reproduced by the filtrate alone. Second, transcriptomic analysis was performed at a single time point, 48 h after treatment. This time point was selected based on morphological and physiological evidence. At 48 h, the mycelia showed obvious shrinkage, swelling, and spore malformation and lysis, whereas earlier time points showed only minor changes. In addition, MDA content, AKP activity, and extracellular macromolecule leakage reached their most pronounced and stable levels at 48 h. These findings indicated that the pathogen had entered an irreversible phase of damage and growth inhibition. Therefore, this time point was chosen to capture terminal transcriptomic features associated with growth inhibition rather than early transient signaling events. However, a single-time-point design cannot resolve early molecular responses occurring within 0–12 h. Because pathogenic fungi can rapidly activate transcriptional defense programs within hours of exposure, future time-series analyses are needed to characterize the dynamic regulatory network underlying the pathogen response. Third, the chemical identities of the bioactive metabolites in the DJ13 fermentation broth have not been characterized. In this study, crude fermentation broth was used instead of purified compounds to define the overall antifungal framework of DJ13 and to evaluate its integrated effects on the pathogen. This approach more closely reflects the natural synergistic action of multiple metabolites in practical biocontrol settings. Moreover, biocontrol agents are typically applied as crude preparations in agricultural practice. Thus, understanding the overall physiological response of the pathogen to a complex metabolite mixture has more direct ecological and practical relevance than examining individual compounds in isolation. Species of the genus *Diaporthe* are known producers of diverse secondary metabolites, including polyketides, alkaloids, terpenoids, and pyrones, many of which have documented antifungal activities [47]. Although the specific compounds responsible for the observed effects in this study remain unidentified, the multi-omics data indicate that the antifungal activity is mediated neither by a single compound nor by a single target, but rather through the synergistic action of multiple metabolites and four pathways: cell membrane damage, cell wall disruption, oxidative stress, and inhibition of energy metabolism. This broad-spectrum mode of action is characteristic of complex microbial secondary metabolite mixtures and supports the potential direct application of the fermentation broth. Nevertheless, identification of the active components remains essential for improving mechanistic understanding and ensuring product quality control. Future studies should combine bioassay-guided fractionation with UHPLC-MS/MS and NMR for systematic metabolite characterization. Fourth, the functional roles of the candidate genes identified in this study remain correlative, and their proposed involvement in the antifungal mechanism requires future validation using functional genetics approaches. Specifically, the up-regulated ABC transporter genes (Cluster-12281.2) may be directly involved in the efflux of antifungal compounds; this hypothesis should be tested by gene knockout or overexpression in *C. jiangxiense*, followed by sensitivity assays against DJ13 fermentation broth. Similarly, cell-wall-related DEGs, such as the up-regulated chitinase and down-regulated α-glucanase genes, need to be functionally characterized through targeted gene disruption or RNA interference to determine whether their altered expression directly causes or contributes to the observed cell wall damage. Furthermore, genes implicated in oxidative stress responses (xanthine dehydrogenase) require genetic manipulation combined with direct ROS measurements to establish a causal link between transcriptional changes and oxidative injury. Despite these limitations, the integrated physiological and transcriptomic evidence provides a coherent and testable mechanistic model and identifies priority pathways and candidate genes for future functional studies.

## 5. Conclusions

By integrating physiological and transcriptomic evidence, we propose a multi-target, multiphasic model for the antifungal activity of DJ13 fermentation broth against *C. jiangxiense*. The primary events involve disruption of the cell membrane and cell wall integrity. Membrane damage was reflected by electrolyte leakage, MDA accumulation, and up-regulation of ABC transporter genes, whereas cell wall disruption was indicated by AKP leakage and transcriptional reprogramming of genes involved in cell wall metabolism. These changes occurred mainly within 12–24 h of treatment. The primary structural injuries were accompanied and amplified by secondary metabolic disturbances, including inhibition of TCA cycle enzymes, energy depletion, ROS-associated oxidative stress, and impaired DNA repair. Transcriptional changes, particularly the up-regulation of efflux transporters, chitinases, and ROS-generating enzymes, appear to reflect the pathogen’s attempt to counter these stresses. However, these responses were insufficient to prevent irreversible cellular damage. This model highlights the potential advantage of crude fermentation broth over purified single compounds, as crude preparations can target multiple cellular processes simultaneously and may reduce the likelihood of rapid resistance development. From a translational perspective, our findings provide a mechanistic rationale for developing DJ13 as a biocontrol agent against rhododendron brown spot. Nonetheless, the mechanism proposed thus far is still a tentative hypothesis based on integrated physiological and transcriptomic evidence and awaits further experimental corroboration. Future studies should focus on formulating bioactive components for field application, evaluating efficacy under greenhouse and field conditions, and performing functional genetic studies to validate the candidate targets identified in this study.

## Figures and Tables

**Table 1:** Data Filtering Statistics.

Sample	Raw Reads	Clean Reads	Q20 (%)	Q30 (%)	GC (%)
CK-1	56,814,350	55,148,174	98.96	95.36	56.35
CK-2	50,856,968	48,998,152	98.88	94.99	56.22
CK-3	57,455,404	55,616,530	99	95.33	56.64
Treat-48h-1	53,355,654	51,200,874	98.96	95.26	56.73
Treat-48h-2	61,017,270	58,611,440	99.08	96.04	56.71
Treat-48h-3	49,726,670	48,345,436	99	95.44	56.73

**Table 2:** Information on DEGs after 48 h of co-culture.

Gene Name	log_2_Fold Treat-48h/CK	Functional Annotation
Cluster-5639.0	2.52	MFS Transporter
Cluster-5171.0	−2.11	Hexose Transporter
Cluster-11741.6	−2.09	ABC Transporter
Cluster-13741.2	3.01	MFS Transporter
Cluster-10380.5	−2.04	MFS Domain-Containing Protein
Cluster-14541.6	−9.69	Encodes Monoacylglycerol Lipase
Cluster-12281.2	7.82	ABC Transporter
Cluster-6458.0	−2.14	Integral Membrane Protein
Cluster-2427.0	−2.69	DASH Family Cryptochrome Protein
Cluster-6516.0	−2.29	DASH Family Cryptochrome Protein
Cluster-1531.0	−3.16	Berberine Bridge Enzyme
Cluster-11963.1	5.45	Aromatic Amino Acid Aminotransferase
Cluster-9813.0	2.41	Xanthine Dehydrogenase
Cluster-13775.0	2.13	L-Pipecolate Oxidase
Cluster-13877.5	2.86	Chitinase
Cluster-4231.0	2.33	Polysaccharide Monooxygenase Cel61a
Cluster-6626.0	−3.09	Transglycosylase
Cluster-14475.3	−10.61	Mannosyl-Oligosaccharide Glucosidase
Cluster-8888.10	−5.95	β-Glucosidase
Cluster-1097.0	3.65	Glucan 1,3-β-Glucosidase
Cluster-5129.0	2.30	Polysaccharide Deacetylase
Cluster-10504.0	3.02	FAD-Dependent Oxidoreductase
Cluster-6309.0	2.04	NADH-Dependent Flavin Oxidoreductase
Cluster-8251.0	2.29	FAD-Dependent Monooxygenase
Cluster-8419.0	2.23	FAD-Dependent Oxidoreductase Superfamily Protein
Cluster-12760.8	−4.88	Trimethyllysine Dioxygenase
Cluster-1884.0	−2.23	Long-Chain Acyl-CoA Synthetase
Cluster-11788.8	−10.70	Oxalate-CoA Ligase

## Data Availability

The raw sequencing data supporting the findings of this study have been deposited in the NCBI Sequence Read Archive (SRA) under the BioProject accession number PRJNA1481915. These data are publicly available and can be accessed through the NCBI SRA database (https://www.ncbi.nlm.nih.gov/bioproject/PRJNA1481915, accessed on 8 July 2026).

## Supplementary Materials

The following supporting information can be downloaded at https://www.mdpi.com/article/10.3390/microorganisms14071530/s1. Table S1: qRT-PCR Primer sequence list.
