# Supplementary material for: Effects of Fermentation Broth from the Biocontrol Fungus Diaporthe novem on Colletotrichum jiangxiense, the Causal Agent of Rhododendron Brown Spot, and Transcriptomic Analysis of the Pathogen
Source: Microorganisms. 2026 Jul 13;14(7):1530. doi: 10.3390/microorganisms14071530 (PMC13414462; doi:10.3390/microorganisms14071530)
Supplement: Supplementary file 1 [file microorganisms-14-01530-s001.zip › microorganisms-4370005-supplementary.pdf]

Table S1 qRT-PCR Primer sequence list

Gene Cluster	Primer sequences (5'–3')
Cluster-5171.0	F：CCTCGGCCGTCGTCTCAC
	R：AGGCGACCAATGGCGAAC
Cluster-14475.3	F：GGCACCACTCCACATT
	R：CCGCTAGAGCCAACAC
Cluster-12760.8	F：CCCATAGTCGGATTCA
	R：TCTCCAGGGCTTTCTT
Cluster-11788.8	F：CGCGAGACCGCAAACT
	R：GCCAGGAACGAGACGA
Cluster-6516.0	F：CAACCACTGCATTTTCTGCG
	R：TCTTTGTCCTCTTGCGTCCC
Cluster-13741.2	F：ACCCGCCAAGGAAACTT
	R：TATGCGCCAGCGAAAAT
Cluster-6516.0	F：CACAAACACCCGTTTCC
	R：TAGCCGTGGGCAGAGAC
Cluster-13741.2	F：GACCGTTCCCCGTCCCT
	R：ATGCCTGGCGTGCTTCC
Cluster-6516.0	F：TACCAACCACGACCCAT
	R：CCAACTTCTTGAACCGC
Cluster-6516.0	F：CGGAGTCAACGGATTTGGTCGTAT
	R：AGCCTTCTCCATGGTGGTGAAGAC
